# Man and His Enemies

**Published:** 1889-06-01

**Authors:** 


### The Doctor's Pulpit.

### MAN AND HIS ENEMIES.

### Home Difficulties.

Man is the most difficult instrument the philosophic musician has to play upon. He can produce music the most varied and the most beautiful, but he can also send forth the most hideous and hateful discords. The term "man" here stands for the race. If it did not, but only for the male animal, we should be tempted to say that there is one creature known to science which can make music still more divine than that of man, and discord still more intolerable, and that creature is woman. It has been said that whatever man can do well woman can do better. That, as a general proposition, is the rankest heresy; but it contains enough of truth to ensure for it a certain degree of currency and acceptance. If it be affirmed that woman can transcend man in many things that are beautiful, and can sink below him in many that are base, we entirely concur. These two wonderfully potential creatures often produce between them an infinite variety of "home difficulties."

It would be a most strange thing if two distinct persons of opposite sexes; each born with a definite individuality; each brought up in a different environment, and taught to look at things from a different standpoint; each with an independent judgment and will; each feeling the necessity of living an individual and complete life—it would be a most strange thing if they could come together as husband and wife, and spend all their days and years without friction and without misunderstanding. As a rule, they cannot do so; it is not in human nature at its present stage of development. But home difficulties sometimes constitute no small part of the dangerous "stress and strain of mid-life." The word "dangerous" is used in this connection, because many people in mid-life are like beleaguered cities. There is a hostile army at every gate. Business may be unprosperous, or at best doubtful; a social standing may have been taken up for which the resources are not quite adequate; health may be uncertain, or frequently giving way; the future on all sides may look like a black and threatening winter storm. If to all these causes of anxiety many and serious "home difficulties" are added, the mid-life of a man or a woman is by no means enviable.

It used to be said that he who made a blade of grass to grow where none grew before, was a benefactor to his kind. In the near future, it will be said, in civilised countries that he who takes away a single anxiety from the mind of over-burdened man is a friend to the race. It is wonderful what brave men can do and endure if they have intermediate periods of complete rest and quiet of mind; if, for example, the home atmosphere be soothing, pleasant, and light; and if at the end of each week there be a complete break in the chain of business, and a reasonable consideration of other questions. But if a man's life consist entirely of days spent in a lion's cage at business, and evenings in a hornet's nest at home, his fate is bad. It may of course be the man's own fault. He may himself be the "blackguard of the village" who incites the lions of the world's menagerie to fury in the daytime, and tumbles over every beehive in the garden the moment he enters his own domain.

But whether man or woman, or both, be at fault, one thing is certain, that an atmosphere of domestic strife and contention is more unfavourable to health in these days of mental stress and strain than bad cooking, bad drainage, or bad air. The interval between business and bedtime should be spent in smoothing out the wrinkles of the mind and stroking down the ruffled coat the right way. A man may purr as well as a cat, and may enjoy it quite as much. The evening, indeed, should be the daily purring time both for man and wife. It is so in the case of young lovers—perhaps of lovers who are no longer very young. They find no difficulty in creating a soothing and delightful atmosphere for each other. What demon of discord is it that so often enters married life and drowns "the Voice that breathed o'er Eden" in a torrent of small contentions and wretched bickerings which are as stupid as they are vicious and miserable?

The doctor can look at questions of this kind entirely from a health point of view. He often sees how sleep, which has been threatening to desert the husband or wife for a long time, is finally driven away by the irritating annoyances that follow the dinner hour; how the remedy he has prescribed for dyspepsia entirely fails because the meal has been made stormy by the selfishness or ill-temper of some member of the family; how the tonic prescription he constructed with so much thought and care is altogether useless, because the mind of the patient never knows ease or quiet. All these, it may be said, are small things; but they are altogether practical and of daily significance. Bread of itself is a small thing, but who can live without it? A potato is an insignificant vegetable; but how much of the comfort and health of the modern civilised man is bound up in the common potato? Life, in a very wide sense, consists in an infinity of small details; and he who has no head for details will often find himself carried far from the main road on the back of some wilful general principle.

The specialist, whatever be his specialty, has to learn his art by constant practice, and by the steady use of his best intelligence. If he neglect either of these conditions, he never learns it at all. The art of "married life" is one of the most difficult specialties a man can have to learn. It is the art of noble conduct in the privacy of home, where no unfriendly critic dare even so much as look. Inasmuch as, from a health point of view alone, this art is one of the most important which man or woman can be called upon to practise, it is essential that it be recognised as a specialty and studied with diligence and conscience. The man, being the head of the house, should feel it incumbent upon himself to be an accomplished specialist, so that both his example and his precepts may have due weight with the rest of the family. But none the less should the woman have a generous ambition to excel, if possible, her husband's most strenuous efforts. This temper of mind, on both sides of the house, will reduce "home difficulties" to a minimum, and will remove one of the most serious obstacles that lie in the way of health, of happiness, and of life success.

---

Vivisection does not seem to be increasing in England. Mr. Erichsen, the inspector, states in his report that 1,069 experiments were performed, being 151 less than last year, and of these only eight were attended with pain to the animals, which were rabbits, guinea-pigs, mice, and frogs. Mr. Erichsen expresses his conviction that those who obtain licenses to perform experiments on live animals are most conscientious in using anæsthetics, in order that the victims may not suffer. English humanity goes hand-in-hand with English science.

